# The role of 18F-FDG PET/CT testing in the management of a papillary thyroid carcinoma

**DOI:** 10.1186/1756-6614-6-S2-A16

**Published:** 2013-04-05

**Authors:** Agnieszka Florczak, Andrea d'Amico

**Affiliations:** 1 Department of Diagnostic PET, Maria Sklodowska-Curie Memorial Cancer Centre and Institute of Oncology, Gliwice Branch, Poland

## Introduction

Thyroid papillary carcinoma represents 75% of all thyroid cancer cases. It is more prevalent in the young and middle age group of patients. Distant metastases occur in 10% of cases. Metastases to lymph nodes can be the first sign of microcarcinoma. This is a case report where thrombophlebitis of popliteal vein and pulmonary embolic disease appeared to be the first manifestation of the carcinoma. The chest CT scan showed mediastinal lymfoadenopathy representing metastases of papillary thyroid cancer. The 18 F-FDG PET/CT scans had a major diagnostic and prognostic value in the overall management.

## Case

A 36 year old man was diagnosed with thrombophlebitis of the right popliteal vein and pulmonary artery disease in September 2011. Chest CT scans showed mediastinal limphoadenopathy. The neck ultrasound imaging was unremarkable. On 29th September 2012 EBUS/TNM confirmed Ca papillare. The 18F-FDG PET/CT scans showed an increasing FDG uptake in the lymph nodes and in the left lobe of thyroid gland. FNAB of 7 mm diameter thyroid nodule detected Ca papillare. In December 2012 a total strumectomy with Transcervical Extended Mediastinal Lymphadenectomy (TEMLA) was performed. The management was complicated by cardiac tamponade. The treatment included 7x metastasectomy as well as 3x 131 I therapy. In the 131 I scintigraphy scan there was no uptake of 131 I. The serum level of Tg was normal. The CT scans suggested recurrence of the carcinoma. In January 2013 18F-FDG PET/CT scans (before the third treatment of 131 I) showed an increasing FDG uptake in several lymph nodes, in the left lung and the subcutaneous lesion of chest.

## Conclusions

The 18F-FDG PET/CT was proved a sensitive and specific imaging modality, useful in assessing the progression of a thyroid papillary carcinoma with suspected recurrences suggested by radiological imaging.

